# The complete chloroplast genome of *Cratoxylum cochinchinense* (Hypericaceae)

**DOI:** 10.1080/23802359.2019.1674216

**Published:** 2019-10-09

**Authors:** Jin Huang, Yachao Wang, Shenjian Xu, Jian He, Zhixiang Zhang

**Affiliations:** a Laboratory of Systematic Evolution and Biogeography of Woody Plants, School of Nature Conservation, Beijing Forestry University, Beijing, China;; b Museum of Beijing Forestry University, Beijing, China

**Keywords:** *Cratoxylum cochinchinense* Blume, complete chloroplast genome, phylogeny

## Abstract

*Cratoxylum cochinchinense* (Lour.) Blume is a tropical Asia specie limited distributed from South China to Borneo. The complete chloroplast genome of the species was found to be 157,103 bp in length, with 129 unique genes, including 37 tRNA, eight rRNA, and 84 protein-coding genes. The GC content of *C. cochinchinense* is 36.2%. As the first complete chloroplast genome in Hypericaceae, it shows the phylogenetic relationship within Malphigiales.

The family Hypericaceae consists of ten genera, more than 500 species (Ruhfel et al. 2011; APG 2016), but no complete chloroplast genome has been published yet. *Cratoxylum* Blume is a native tropical Asia genus including about six species (Gogelein 1967). *Cratoxylum cochinchinense* (Lour.) Blume is a kind of deciduous flowering shrub or tree, widespread from South China to Borneo. Its stem can be carved with the hard, delicate texture. Young fruit was usually used for cooking spices, and tender leaves can be used as substitutes of tea (Li et al. 1997). The first plastome sequence of *C. cochinchinense* was reported in this study.

Fresh leaves of *C. cochinchinense* were collected from Houmiling Provincial Forest Park, Dongfang city, Hainan Province, China. The voucher specimen was deposited in Museum of Beijing Forestry University, BJFC (specimen No.: Z.X. Zhang zzx2017120806). The total genomic DNA was extracted from the silica gel dried leaf by CTAB method (Doyle and Doyle 1987) with minor modification and then sent to Novogene Company (http://www.novogene.com, China) for next-generation sequencing using Illumina Hiseq 4000 platform. About 5 Gb high quality, 2 × 150 bp PE reads were obtained from high-throughput sequencing. Referring to *Garcinia mangostana* L., we filtered available reads using the Map tool of Geneious R11 (Kearse et al. 2012), these reads were used for de novo assembling. Gaps were bridged by Fine Tuning function. Plann (Huang and Cronk 2015) was employed to annotate the chloroplast genome sequence, and modification was also performed by Geneious R11.

The total length of the chloroplast genome is 157,103 bp. It consists of a pair of inverted repeat regions (IRs) of 26,272 bp, a large single copy region (LSC) of 85,664 bp, and a small single region (SSC) of 18,895 bp. The overall GC-content of the whole pastime genome is 36.2%, while that in LSC, SSC, and IR regions is 34.0%, 29.9%, and 42.1%, respectively. The sequence contained 129 unique genes, including 37 tRNA, eight rRNA, and 84 protein-coding genes.

To confirm the phylogenetic relationship of *C. cochinchinense* in Malphigiales, a Bayesian Inference (BI) tree was constructed using MrBayes 3.2.6 (Ronquist and Huelsenbeck 2003), based on 17 published complete chloroplast genome sequences of Malphigiales and two more sequences that *Averrhoa* in Oxalidales and *Euonymus* in Celastrales are used as outgroup. All sequences were aligned using MAFFT v7 (Katoh and Standley 2013). As shown, *Cratoxylum* in Hypericaceae and *Garcinia* in Clusiaceae were clustered into a clade perfectly, which is sister to *Chrysobalanus* and *Hirtella* in Chrysobalanaceae (Li et al. 2019). It further clarifies the phylogenetic relationship among the generalised Clusiaceae. The plastome sequence of *C. cochinchinense* will be an important reference for the phylogenetic and evolutionary studies of Hypericaceae and Malphigiales.

## Figures and Tables

**Figure 1.**
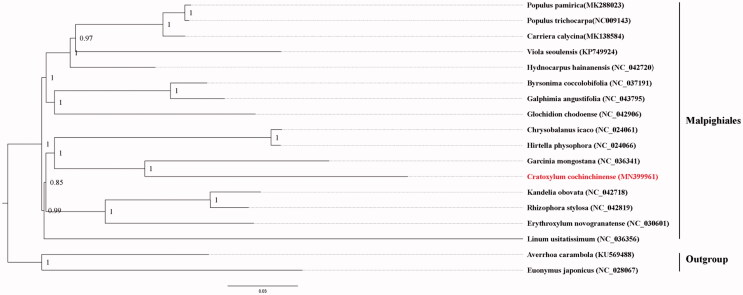
Bayesian phylogram of *Cratoxylum cochinchinense* as well as *Malphigiales* species inferred from the complete plastome sequences.
